# Comprehensive molecular and morphological resolution of blubber stratification in a deep-diving, fasting-adapted seal

**DOI:** 10.3389/fphys.2022.1057721

**Published:** 2022-12-14

**Authors:** J. I. Khudyakov, K. N. Allen, D. E. Crocker, N. S. Trost, A. H. Roberts, L. Pirard, C. Debier, E. R. Piotrowski, J. P. Vázquez-Medina

**Affiliations:** ^1^ Department of Biological Sciences, University of the Pacific, Stockton, CA, United States; ^2^ Department of Integrative Biology, University of California, Berkeley, Berkeley, CA, United States; ^3^ Department of Biology, Sonoma State University, Rohnert Park, CA, United States; ^4^ Louvain Institute of Biomolecular Science and Technology, Université Catholique de Louvain, Louvain-la Neuve, Belgium

**Keywords:** blubber depth, transcriptome (RNA-seq), histology, seal (pinnipedia), metabolism and endocrinology, adipogenesis

## Abstract

Blubber is a modified subcutaneous adipose tissue in marine mammals that provides energy storage, thermoregulation, hydrodynamic locomotion, and buoyancy. Blubber displays vertical stratification by lipid content, fatty acid composition, and vascularization, leading to the assumption that deeper blubber layers are metabolically active, while superficial layers are mainly structural and thermoregulatory. However, few studies have examined functional stratification of marine mammal blubber directly, especially in pinnipeds. We characterized morphological and transcriptional differences across blubber layers in the northern elephant seal, a deep-diving and fasting-adapted phocid. We collected blubber from seals early in their fasting period and divided blubber cores into three similarly sized portions. We hypothesized that the innermost blubber portion would have higher 1) heterogeneity in adipocyte size, 2) microvascular density, and 3) expression of genes associated with metabolism and hormone signaling than outer blubber. We found that adipocyte area and variance increased from outermost (skin-adjacent) to innermost (muscle-adjacent) blubber layers, suggesting that inner blubber has a higher capacity for lipid storage and turnover than outer blubber. Inner blubber had a higher proportion of CD144+ endothelial cells, suggesting higher microvascular density. In contrast, outer blubber had a higher proportion of CD4+ immune cells than inner blubber, suggesting higher capacity for response to tissue injury. Transcriptome analysis identified 61 genes that were differentially expressed between inner and outer blubber layers, many of which have not been studied previously in marine mammals. Based on known functions of these genes in other mammals, we suggest that inner blubber has potentially higher 1) adipogenic capacity, 2) cellular diversity, and 3) metabolic and neuroendocrine signaling activity, while outer blubber may have higher 1) extracellular matrix synthesis activity and 2) responsiveness to pathogens and cell stressors. We further characterized expression of nine genes of interest identified by transcriptomics and two adipokines with higher precision across blubber layers using targeted assays. Our study provides functional insights into stratification of blubber in marine mammals and a molecular key, including CD144, CD4, *HMGCS2*, *GABRG2*, *HCAR2*, and *COL1A2*, for distinguishing blubber layers for physiological and functional studies in seals.

## Introduction

Adipose tissue, which stores energy, provides insulation, and regulates metabolic and immune homeostasis, in part by producing adipokine hormones, has been crucial for mammalian evolution. It has supported adaptations such as migration, heterothermy, and lactation, and has enabled colonization of new environments and diet specialization (Pond, 2012). For example, large adipose tissue stores modified as blubber enabled mammals to recolonize marine environments (Liwanag et al., 2012). Blubber, which can comprise up to 50% of the body mass of some marine mammals, plays a vital role in energy storage, thermoregulation, hydrodynamic locomotion, and buoyancy (Iverson, 2009). While adipose tissue in terrestrial mammals is organized in site- and function-specific depots, the majority of body fat in marine mammals is localized in a somewhat uniform subcutaneous blubber layer comprised of white adipocytes (and potentially, some brown or beige adipocytes; Hashimoto et al., 2015) embedded in a network of structural protein fibers (Iverson, 2009). At present, the functional, molecular, and morphological differences between blubber of marine mammals and adipose tissue of other mammals are not fully understood.

Numerous studies of marine mammal blubber, particularly in cetaceans, have shown vertical stratification of this tissue by lipid content, fatty acid composition, degree of vascularization, and concentration of lipophilic hormones and pollutants (Derous et al., 2020). While the degree of stratification varies by species, the inner blubber layer of most marine mammals contains a larger proportion of dietary fatty acids, especially saturated and polyunsaturated fatty acids, is more highly vascularized, and has higher concentrations of hormones than the outer layer (Strandberg et al., 2008; McClelland et al., 2012; Fowler et al., 2014; Waugh et al., 2014; Louis et al., 2015; Guerrero and Rogers, 2017; Kershaw et al., 2017; Kershaw et al., 2019; Gabler-Smith et al., 2022). These findings have led to the assumption that the inner blubber layer is more metabolically active, while the outer layer serves mainly structural and thermoregulatory roles. Therefore, the different blubber layers in marine mammals may be analogous to distinct adipose tissue depots in terrestrial mammals.

However, few studies have examined the functional stratification of marine mammal blubber directly. One found that genes encoding the adipokine leptin, the leptin receptor, and two lipases were expressed more highly in inner compared to outer blubber of beluga and bowhead whales (Ball et al., 2017). Another showed that *ex vivo*-cultured slices of inner blubber tissue of northern elephant seals released more leptin hormone in response to lipolytic stimuli than slices of outer blubber. In addition, glycerol release in response to lipolytic stimuli decreased between early and late fasting in inner blubber slices, whereas it remained constant in outer blubber (Debier et al., 2020). Further functional studies of marine mammal blubber are clearly needed, as this tissue may provide important insights into evolution, health, and conservation of marine mammals, as well as metabolic disease in humans (Derous et al., 2020). Such studies may be more practical in pinnipeds than cetaceans due to their smaller body sizes and accessibility on land or ice. However, aside from fatty acid composition (e.g., Tverin et al., 2019), blubber stratification has not been extensively studied in this clade, confounding the results of physiological studies utilizing this tissue.

The northern elephant seal has been used in such physiological studies for decades (Houser et al., 2013). Based primarily on fatty acid stratification data from this and other pinniped species (Louis et al., 2014), we typically use the inner half of blubber biopsies collected from elephant seals in our analyses of blubber responses to physiological stressors (Deyarmin et al., 2019; Khudyakov et al., 2022). However, the physical boundary between these layers is not visually apparent, and blubber from elephant seals has not been included in previous morphological studies (Gabler-Smith et al., 2022). Therefore, we aimed to 1) characterize morphological differences between blubber layers using histology and immunohistochemistry, 2) identify the molecular signatures that distinguish inner from outer blubber using transcriptomics, and 3) use these signatures to precisely determine the boundary between blubber layers using RT-qPCR in the northern elephant seal. We hypothesized that inner blubber would have higher heterogeneity in adipocyte size, be more vascularized, and have higher expression levels of genes associated with metabolism and hormone signaling than outer blubber. The findings of our study provide functional insights into stratification of blubber in marine mammals and a molecular key for distinguishing blubber layers for physiological and functional studies in seals.

## Methods

### Reagents

All reagents were purchased from Fisher Scientific or VWR (United States), unless otherwise indicated.

### Sample collection

Animal sampling was conducted under National Marine Fisheries Service permit 19108 at Año Nuevo State Reserve (San Mateo County, CA, United States). All animal handling procedures were approved by the Sonoma State University Institutional Animal Care and Use Committee. For histological analyses, five juvenile northern elephant seals (three females, two males) were sampled in December 2019. For transcriptome analyses, three female juvenile northern elephant seals were sampled in January 2020. Juvenile animals were presumed to be early in their fasting period based on body condition, although the exact duration of fasting prior to sampling was unknown. For qPCR analyses, five weaned pups (two females, three males) were sampled in February–March 2022. Pups were sampled early in their post-weaning fast, which was determined by presence of unmolted natal pelage and body condition. Different age classes were used due to their availability at the rookery at the time of sampling for the project.

**FIGURE 1:**
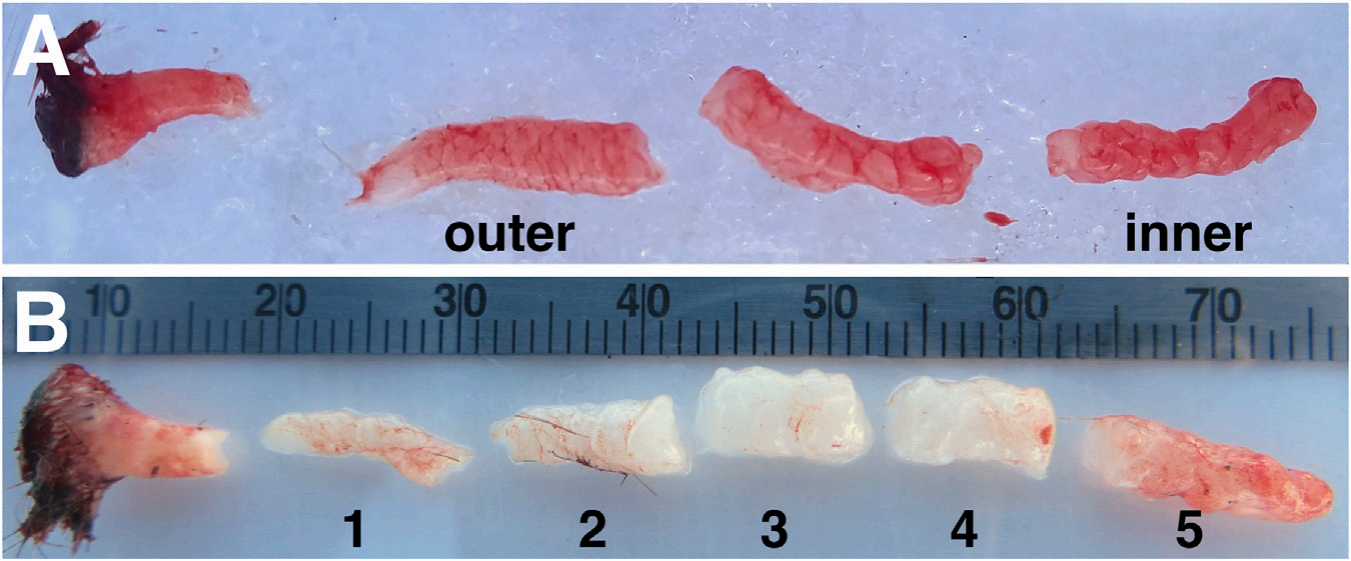
Example of northern elephant seal blubber biopsies used for **(A)** transcriptome analyses and **(B)** targeted gene expression assays. The former was collected from juvenile elephant seals and dissected into three layers; the innermost and outermost layers were used for transcriptomics. The latter was collected from weaned elephant seal pups and dissected into five ∼1-cm layers (numbered 1–5 from outer-to innermost). Differences in tissue color are due to differences in blubber perfusion in individual animals at time of sampling.

Animals were sedated and sampled as described previously (Debier et al., 2020; Pujade Busqueta et al., 2020). Briefly, seals were chemically immobilized using an intramuscular injection of ∼1 mg/kg tiletamine-zolazepam HCl (Telazol, Fort Dodge Animal Health, United States), and sedation was maintained with intravenous doses of ketamine (0.25–1 mg/kg) (Fort Dodge Animal Health, IA, United States). Blubber biopsies (full cores including skin and muscle) were collected from the posterior flank of each animal using a 6.0 mm diameter biopsy punch (Miltex Integra, United States). For histology, biopsy samples were placed in 10% neutral-buffered formalin for 24 h at 4°C, washed three times in PBS, and stored in 70% ethanol until further processing. For transcriptome profiling, biopsies were dissected on ice into three similar-sized blubber portions after removing skin and muscle, as the boundary between inner and outer blubber is not visually apparent (Figure 1A). For gene expression profiling by RT-qPCR, blubber biopsies were dissected into five (approx. 1-cm) pieces to obtain higher resolution of the inner-outer blubber boundary (Figure 1B). For transcriptome and qPCR analyses, blubber samples were frozen immediately after dissection on dry ice and stored at −80°C until further processing.

### Histology and adipocyte area measurements

Fixed tissues were dehydrated, embedded in paraffin, sectioned longitudinally into 5-μm thick sections (one blubber sample per slide), and stained for hematoxylin and eosin (H & E) using standard protocols at UCSF Gladstone Histology and Light Microscopy Core. H & E-stained sections were imaged on a Zeiss Axio Scan Z1 microscope fitted with a Plan Apochromat 20X/0.8 M27 objective. Images of sections were divided into three equal-sized portions (outer, middle, and inner blubber “layers”) and cross-sectional areas of adipocytes were quantified in at least two regions of interest (ROIs; minimum 40 cells/ROI, Table 2) within each blubber layer using the FIJI plugin Adiposoft (v1.16). Only cells with diameters 70–800 µm were measured, and Adiposoft results were manually corrected to avoid inclusion of partial cells in the counts. Sections from one animal were excluded from analyses due to inadequate quality for area measurement.

### Immunofluorescence

Unstained tissue sections were deparaffinized, rehydrated using xylene and ethanol, permeabilized, blocked, and incubated overnight at 4°C with primary antibodies (CD4, R&D Systems, Cat. No. MAB2410, 25 μg/ml; CD144, Thermo Fisher, Cat. No. PA5-19612, 1:100) as previously described (Vázquez-Medina et al., 2016). Sections were washed and incubated with Alexa Fluor 594-specific secondary antibodies diluted 1:500. Nuclei were counterstained with SYTOX green. Sections were mounted using Vectashield Antifade Mounting Medium (Vector Laboratories, United States) and imaged using a Zeiss LSM 780 AxioExaminer microscope fitted with a 20X water-immersion objective. CD4-positive cells were counted using Fiji-ImageJ in at least six different fields per section (for both inner and outer blubber), per slide, and per individual. Results are expressed as average positive cells per field. Vascular density (Vessel Area Fraction, VAF) was evaluated in sections stained with CD144 using the Vascular Density FIJI plugin (Corliss et al., 2019).

### RNA isolation

Blubber samples were minced with a scalpel on dry ice and homogenized in 1 ml Qiazol (Qiagen, United States) by bead beating using a Bullet Blender Storm 24 (Next Advance, United States) as described previously (Pujade Busqueta et al., 2020). Homogenates were further disrupted using a 21-gauge needle and syringe. After phase extraction with chloroform, RNA was purified from the aqueous phase using RNeasy Lipid Mini Kit (Qiagen, United States) following the manufacturer’s protocol. An on-column DNase I digest was used to remove genomic DNA. RNA quantity and integrity were determined using the High Sensitivity RNA Assay on the Qubit 3.0 Fluorometer (Life Technologies, United States) and the RNA Pico 6,000 Assay on the 2,100 Bioanalyzer (Agilent Technologies, United States), respectively. Samples used for RNAseq had RIN values of 7.7—8.8.

### Transcriptome sequencing and annotation

Strand-specific 150-bp paired-end cDNA library preparation and NovaSeq S4 sequencing were conducted by QB3 Genomics, UC Berkeley, Berkeley, CA (RRID:SCR_022170). Raw data were uploaded to NCBI Sequence Read Archive (BioProject ID: PRJNA874098). Transcriptome assembly, annotation, and transcript abundance estimation were conducted using the Extreme Science and Engineering Discovery Environment (XSEDE) Bridges2 Large High Performance Computing Cluster at the Pittsburg Supercomputing Center (allocation TG-IBN150010; Towns et al., 2014). The transcriptome was assembled *de novo* using the default Trinity v2.11.0 pipeline (Haas et al., 2013). Assembled transcripts were annotated by blastx against the UniProt Caniformia database (Taxonomy ID: 379584, 549,465 sequences, downloaded 8/10/2021) using Diamond v0.8.31 run in ultra-sensitive mode (k = 1, e-value = 1e-3; Buchfink et al., 2015). Differential gene expression analyses were conducted using DESeq2 (see “Statistical Analyses” section below).

### RT-qPCR

Reverse transcription (RT) was performed using an input of 500 ng total RNA with the SuperScipt IV VILO kit with ezDNase (Thermo Fisher, United States). cDNA samples were diluted 1:10 and 2 μl were used in each 20-μl qPCR reaction using PowerUP SYBR Green Master Mix (Thermo Fisher, United States). qPCR was performed on a QuantStudio 5 Real-Time PCR System instrument (Thermo Fisher, United States) using the following program: 2 min at 50°C and 2 min at 95°C, followed by 40 cycles of 15 s at 95°C and 60 s at 60°C. All primers were used at 400 nM final concentration. All samples were run in triplicate with all intra-assay and inter-assay CVs <0.6%. No-template and no-RT enzyme controls were included in each run and showed no amplification.

Primers for qPCR (Table 1) were designed to target highly conserved regions of differentially expressed genes of interest using PrimerQuest Tool (Integrated DNA Technologies, United States). Candidate genes were selected based on significant blastx hits to a protein with known function in other species and high transcript expression in elephant seal blubber (transcript per million, TPM >100). Primer efficiencies (Table 1) were determined using standard curves of four 1:2 dilutions of pooled cDNA. Primer specificity was confirmed using melt curve analysis and gel electrophoresis. Normalized gene expression values (delta C_T_) were obtained by subtracting the cycle threshold (C_T_) of genes of interest from the geometric average of the C_T_s of YWHAZ and NONO, which have been previously validated for use as reference genes in elephant seal blubber (Pujade Busqueta et al., 2020). The CV for C_T_s of YWHAZ and NONO across all samples in this study were 2.09% and 2.44%, respectively. YWHAZ and NONO C_T_s did not vary by blubber layer or by study animal (*p* > 0.05). LEP and ADIPOQ primers were described and validated previously (Khudyakov et al., 2019). Mean C_T_s for all samples and primer sets used in the study are shown in Supplementary Material S2.

**TABLE 1:** Sequences and amplification efficiencies of primers used in the study.

Target gene	F. Primer sequence	R. Primer sequence	Efficiency (%)
*AGT*	CAG​ACT​CGG​AAA​GGT​GCT​AAA	CTC​GTA​AAT​GGC​AAA​CAG​GAA​C	94.7
*ANGPTL4*	TCA​GAT​GGA​GGC​TGG​ACT​ATA​A	CAC​CTT​GAG​GGT​CTC​CAA​AG	104.3
*CD4*	CCT​TCA​CCT​TGG​AGA​ACA​AGA​A	GAT​AAA​GCT​GAG​CGG​GAG​AAA	96.9
*CES1*	TGG​TAT​TTG​GTG​TCC​CAT​CTG	GAC​GAG​AAG​CTT​GGA​CGA​TAC	99.3
*COL1A2*	CCC​TAA​CCA​AGG​ATG​CAC​TAT​G	CAG​TTC​TTG​GCT​GGG​ATG​TT	95.6
*GABRG2*	ACC​ATT​GAT​ATT​CGC​CCA​AGA	TTG​CCA​TCC​AGA​CAC​TCA​TAC	91.3
*HCAR2*	GTG​TTC​CGG​GAT​GAC​TTC​ATA​G	TTT​CCA​GGA​CTT​GAG​GTG​AAA​G	100.0
*HMGCS2*	TGA​TGT​TCA​GTG​ACT​TCC​TGT​C	TGT​AGG​TTT​CTT​CCA​GCG​TTA​G	107.3
*LEP*	ACA​GGA​CCA​AAG​CCA​CAG​GA	GCG​AGG​CCT​GAG​AAG​CAC​AT	104.5
NONO*	GAG​GAA​GGT​TTC​GGA​CTG​TAA​G	GCG​GAG​ATT​GCC​AAA​GTA​GA	94.9
*THBS1*	TGA​CTC​AGG​ACC​CAT​CTA​TGA	TTT​CAG​GTC​GGA​GAA​GAA​CAC	100.0
YWHAZ*	AGC​AGA​GAG​CAA​AGT​CTT​CTA​TT	GAC​TGA​TCC​ACA​ATC​CCT​TTC​T	100.3

All sequences are in the 5′ to 3′ direction. * denotes reference genes.

### Statistical analyses

All statistical analyses were conducted using R v4.1.0 (R Core Team, 2019), unless otherwise indicated. For morphological analyses, differences in adipocyte area among blubber regions (innermost, middle, outermost) were assessed for each individual seal using a Kruskal-Wallis test with post-hoc pairwise Wilcoxon rank sum tests, as the data did not meet assumptions of parametric tests. Homogeneity of variance in adipocyte areas among blubber regions was assessed for each individual seal using Brown-Forsythe test with post-hoc pairwise tests using the “onewaytests” package (Dag et al., 2018). All *p*-values for pairwise tests were corrected using a Benjamini–Hochberg adjustment for multiple hypothesis testing. Immunofluorescence data were analyzed using paired t-tests (inner vs. outer blubber), with significance threshold set to *α* = 0.05.

For transcriptome analyses, transcript abundance was estimated using Salmon v1.5.2 (Patro et al., 2017) using XSEDE and summarized using tximport v1.20.0 in R. Differential expression analyses were conducted using DESeq2 v1.32.0 (Love et al., 2014). After filtering out transcripts with low expression levels (counts ≤10 in ≥3 samples), abundance of the 120,277 remaining transcripts was compared between blubber layers (inner, outer) with blocking by individual (model design = ∼ subject + type), with alpha = 0.05, and log2 fold change threshold = 1. Differentially expressed genes that had no blastx hits to the UniProt SwissProt or GenBank non-redundant (nr) protein sequences database (e-value threshold = 1e-3) were annotated by blastn against the GenBank reference RNA sequences (refseq_rna) database (e-value threshold = 1e-10). DESeq2 output for differentially expressed genes is shown in Supplementary Material S1.

For qPCR analyses, differences in normalized gene expression values (delta C_T_s) between blubber layers were assessed using linear mixed-effects models with blubber slice as a fixed effect and animal ID as a random effect using lme4 and lmerTest (Bates et al., 2015; Kuznetsova et al., 2017). Levene’s and Shapiro-Wilk’s tests were used to determine whether variables and model residuals met equal variance and normality assumptions, respectively. Post-hoc comparisons between blubber layers were conducted using estimated marginal means (EMM) with the emmeans package (adjustment = Tukey; Lenth, 2021).

## Results

We collected blubber tissue from juvenile elephant seals early in their fasting period, dissected it into three equally-sized layers (Figure 1A), and compared the morphology and transcriptomes of the blubber layers.

**FIGURE 2:**
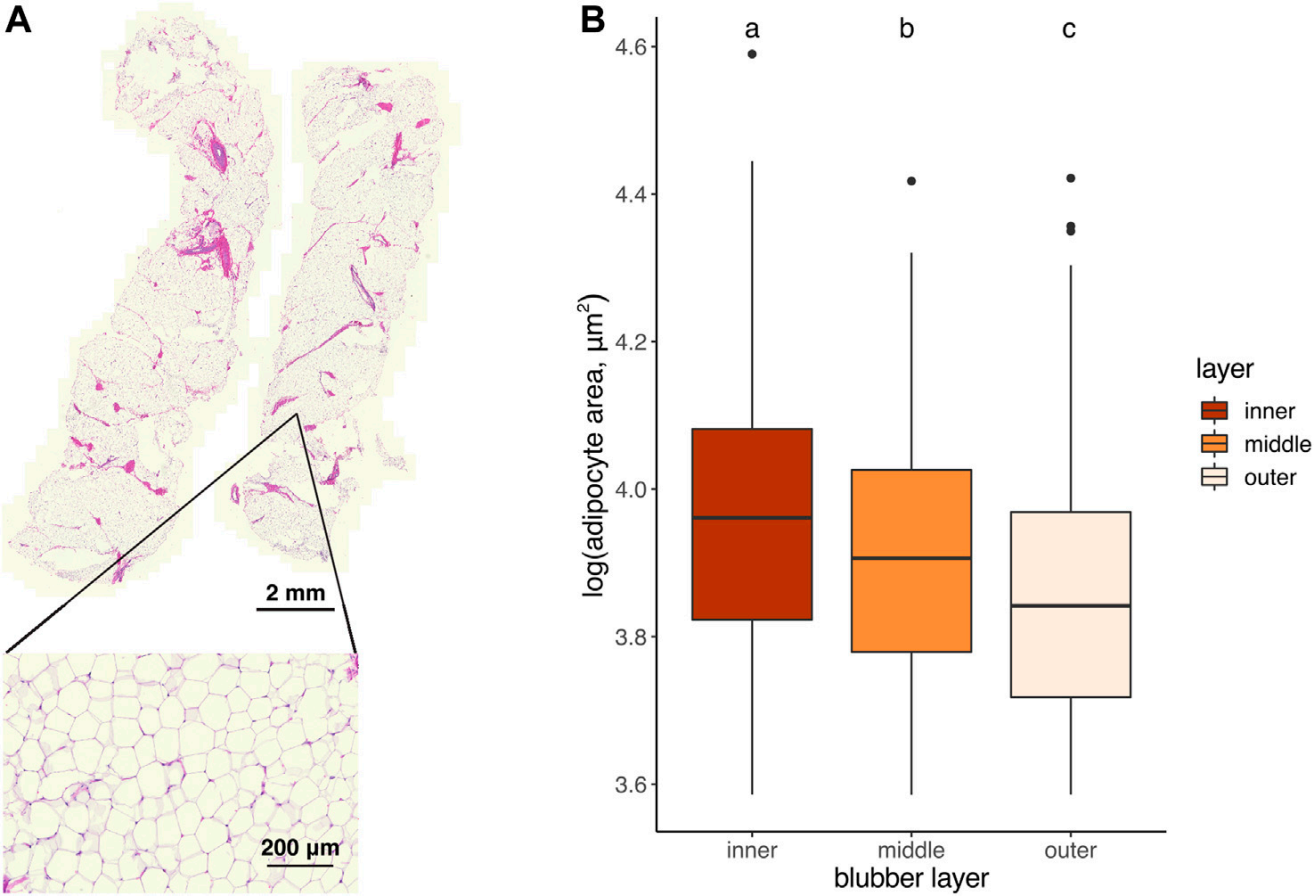
**(A)** Longitudinally-sectioned elephant seal blubber sample stained with hematoxylin and eosin. Inset shows an example region of interest (ROI) within which adipocyte areas were measured. **(B)** Cross-sectional area of adipocytes in outer, middle, and inner blubber regions of four juvenile elephant seals. Different letters denote values that were significantly different (*p* < 0.05) between blubber regions.

**FIGURE 3:**
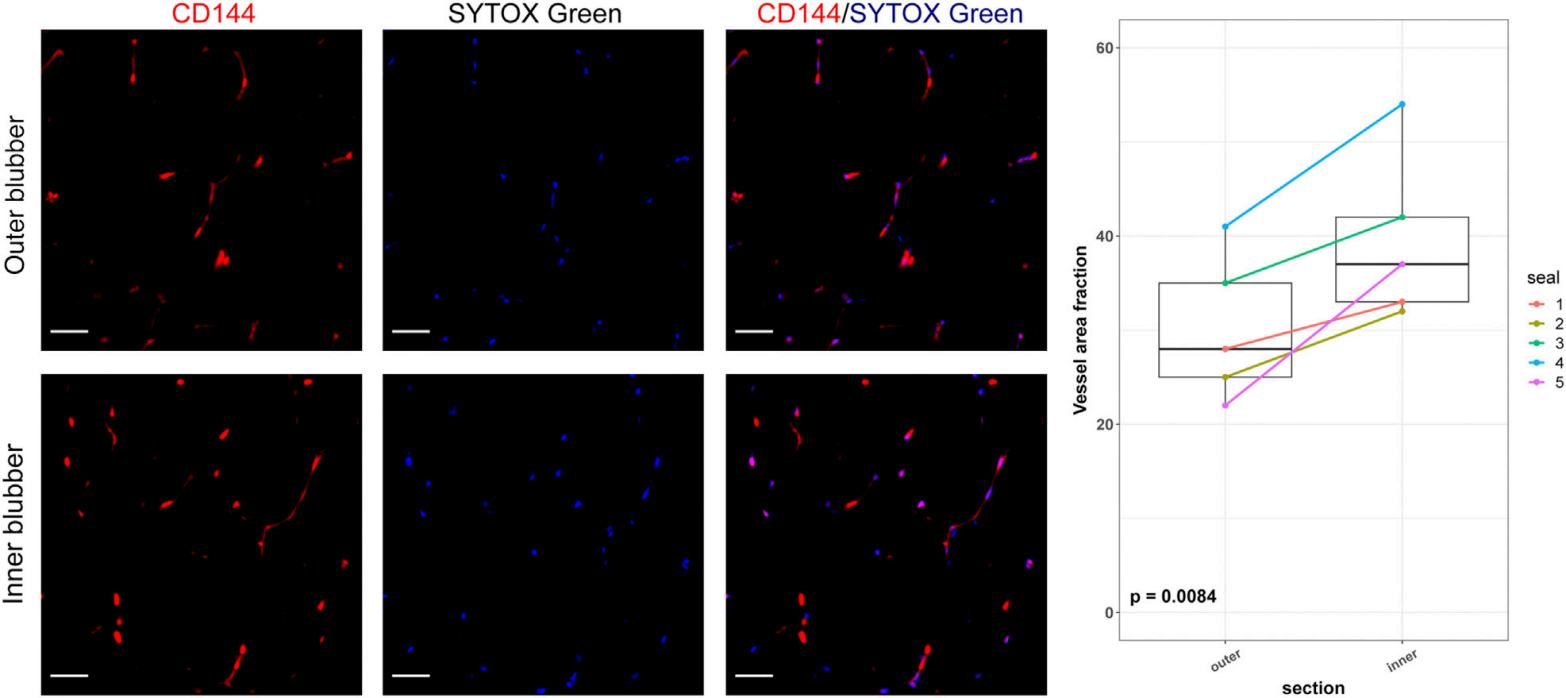
Vascular area fractions in inner and outer blubber tissue layers. Sections of inner and outer blubber of juvenile northern elephant seals (*n* = 5) were stained with CD144 (vascular endothelial cadherin) antibody. Nuclei were counterstained with SYTOX Green. Vascular area fractions were compared between blubber layers using paired t-test. Scale bar is 50 μm.

To determine whether adipocyte size varied by blubber depth, we measured the cross-sectional area of a total of 4,195 adipocytes in inner, middle, and outer blubber layers collected from four seals (Table 2; Figure 2A). Adipocyte area was significantly different between blubber layers (*F* (25,125) = 36.62, *p* < 0.0001; Figure 2B). Adipocyte area was highest in inner blubber (mean ± s.d. = 9,715 ± 3,996 μm^2^), followed by middle blubber (8,578 ± 3,242 μm^2^), and lowest in outer blubber (7,732 ± 3,251 μm^2^; post-hoc pairwise tests, *p* < 0.0001; Table 2). We next assessed whether heterogeneity in adipocyte area varied with blubber depth. Variance in adipocyte area was not equal between blubber layers (*F* (22,188) = 86.22, *p* < 0.0001); it was highest in inner blubber, followed by outer blubber, and was lowest in middle blubber (post-hoc pairwise tests, *p* < 0.0001). Lastly, we assessed whether microvascular density varied between inner and outer blubber by immunolabeling sections of blubber tissue with CD144 (vascular endothelial cadherin) antibody. The vessel area fraction, or fraction of an image composed of blood vessels, was higher in inner compared to outer blubber layers (*t* = 4.85, *p* = 0.0084; Figure 3).

**TABLE 2:** Number of adipocytes measured (*n*) and mean (±s.d.) cross-sectional areas of adipocytes in outermost (skin-adjacent), middle, and innermost (muscle-adjacent) blubber regions of juvenile northern elephant seals (*n* = 4).

Seal	Region	*n*	Mean cross-sectional area (s.d.; μm^2^)
1	Outer	114	8,238 (3,212)
	Middle	368	8,054 (2,789)
	Inner	307	9,586 (3,731)
2	Outer	137	6,823 (3,176)
	Middle	503	8,325 (3,333)
	Inner	857	9,001 (3,259)
3	Outer	106	8,734 (3,662)
	Middle	605	9,105 (3,437)
	Inner	809	10,863 (4,558)
4	Outer	82	7,255 (2,268)
	Middle	200	8,588 (2,951)
	Inner	107	7,123 (2,814)

**FIGURE 4:**
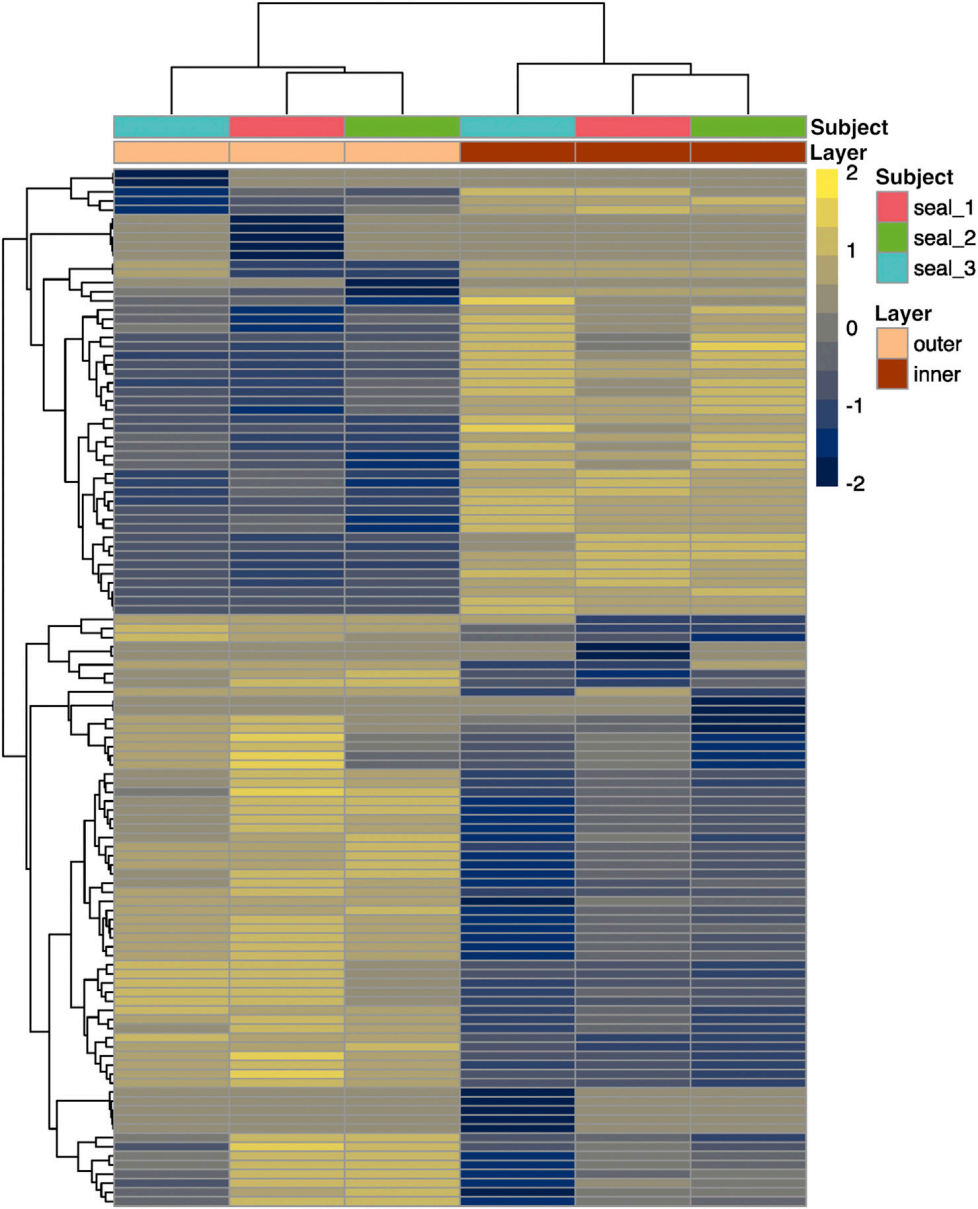
Heatmap showing scaled relative expression levels (transcripts per million, or TPM) of 61 annotated genes that were differentially expressed between inner and outer layers of blubber in juvenile northern elephant seals (*n* = 3). Genes with higher expression in inner blubber are shown in yellow, while those with higher expression in outer blubber are shown in blue. Rows and columns were clustered by expression similarity (Euclidean distance).

We then examined differences in transcriptome profiles between blubber layers. We sequenced the transcriptome of inner and outer blubber layers and assembled 930,306 transcripts, of which 114 were differentially expressed between inner and outer blubber (Figure 4). Forty-nine genes were upregulated in inner compared to outer blubber, of which 33 had unique hits to annotated genes in other species (Supplementary Material S1). Manual UniProt database search was used to infer the functions of inner blubber-enriched genes. They included four members of the Wnt signaling pathway (*WNT6*, *FRZB*, *DKK2*, *SFRP1*) and genes associated with functions such as lipid catabolism (*HMGCS2*, *CES1*), regulation of adiponectin secretion (*HCAR2*, *CCK*), cell survival (*BCL2A1*), regulation of angiogenesis (*THBS1*, *ANGPTL4*), response to GABA neurotransmitter (*GABRG2*), cell-cell and cell-extracellular matrix (ECM) interaction (*LAMC3*, *CCDC125*, *PPFIA2*, *CRYBG2*, *NEBL*, and *EPCAM*), regulation of gene expression (*PRDM7*, *RBPMS*), protein translation (*RPL12*), protein ubiquitination (*LNX1*), protein and amino acid catabolism (*PRSS12*, *XPNPEP3*, *UROC1*), xenobiotic metabolism (*AOX2*), collagen fibril organization (*FMOD*), transmembrane ion transport (*LRRC38*, *STAC2*, and *CNBG1*), signal transduction (*ZNF720*), and zinc ion binding (*CA8*).

Sixty-five genes were upregulated in outer relative to inner blubber, of which 28 had unique hits to annotated genes in other species (Supplementary Material S1). These included two collagen isoforms (*COL1A1*, *COL1A2*), angiotensinogen (*AGT*), and genes associated with immune function (*IL1RL1*, *CD4*, and *CFB*), lipid metabolism (*CYP1B1*), response to cell stressors such as DNA damage (*PBK*, *PARP4*) and protein misfolding (*HSPE1*, and *CREB3L1*), neuronal axon guidance (*EPHB2*, *NTRK2*), protein secretion (*MIA2*, *RAB3IL1*), mitotic spindle assembly (*KIF23*, *BUB1B*, and *STMN1*), protein ubiquitination and degradation (*TMPRSS11D*, *UBXN2A*), regulation of gene expression (*ZNF469*, *GRSF1*), calcium ion binding (*CALR3*), cell-ECM adhesion (*GPC1*), gap junctions (*GJA1*), ER function (*SEZ6L*), G protein-coupled receptor signaling (*ADGRD1*), and blood clotting (*F13A1*).

Lastly, we aimed to further delineate the boundary between inner and outer blubber using targeted assays (RT-qPCR) of differentially expressed genes of interest. Target genes were selected based on high-confidence hits to genes with known functions of interest (e.g., lipid metabolism) in other species and high expression levels in the transcriptome. We selected three genes with higher expression in outer than inner blubber (*AGT*, *CD4*, and *COL1A2*), six genes with higher expression in inner than outer blubber (*ANGPTL4*, *CES1*, *GABR2*, *HCAR2*, *HMGCS2*, *THBS1*), and genes encoding the adipokines leptin (*LEP*) and adiponectin (*ADIPOQ*), which we identified in previous studies. We collected blubber from five recently weaned elephant seal pups (early in their fasting period), divided the tissue into five 1-cm long layers (Figure 1B), and compared gene expression across the five blubber layers.

**FIGURE 5:**
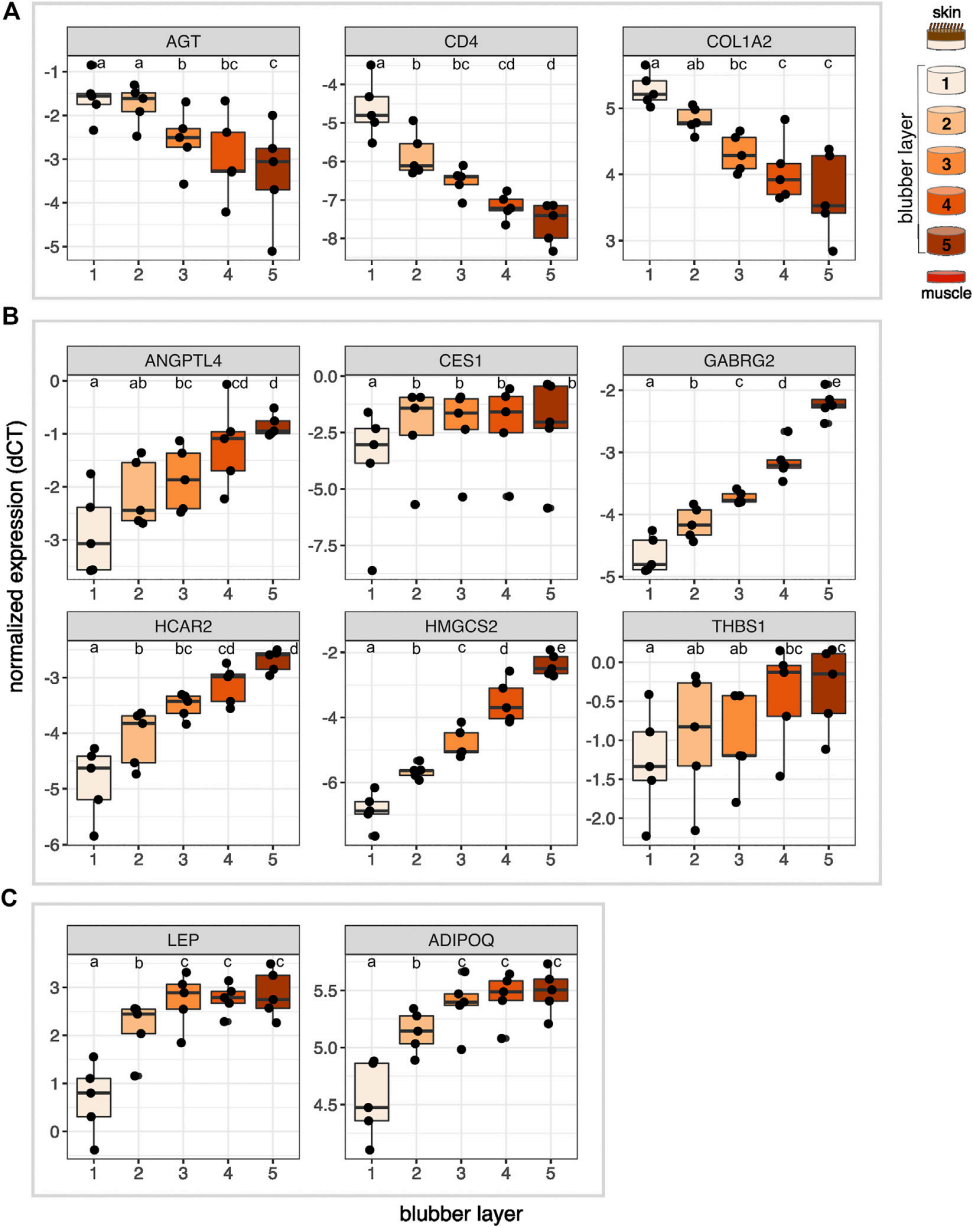
Normalized expression (delta C_T_) of 11 genes of interest across five layers of blubber of northern elephant seal pups (*n* = 5). Target genes were **(A)** identified as upregulated in outer blubber, **(B)** identified as upregulated in inner blubber by transcriptomics, and included **(C)** two adipokine genes of interest. Different letters denote expression levels that were significantly different (*p* < 0.05) between layers. *AGT*, angiotensinogen; *CD4*, cluster of differentiation 4; *COL1A2*, collagen alpha-2(I) chain; *ANGPTL4*, angiopoietin-related protein 4; *CES1*, carboxylesterase 1; *GABRG2*, gamma-aminobutyric acid receptor subunit gamma-2; *HCAR2*, hydroxycarboxylic acid receptor 2; *HMGCS2*, hydroxymethylglutaryl-CoA synthase, mitochondrial; *THBS1*, thrombospondin-1; *LEP*, leptin; *ADIPOQ*, adiponectin.

**FIGURE 6:**
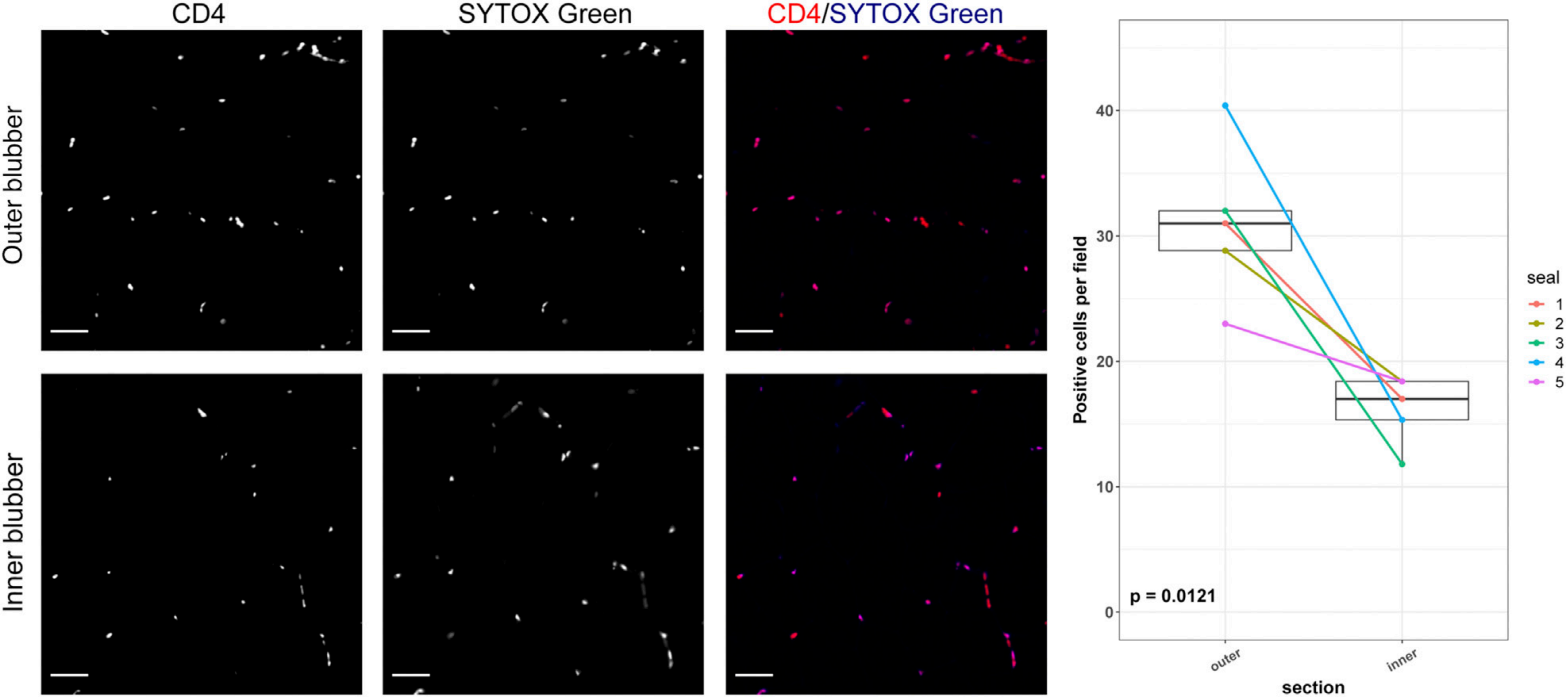
CD4-positive cells in inner and outer blubber tissue layers. Sections of inner and outer blubber of juvenile northern elephant seals (*n* = 5) were stained with CD4 antibody. Nuclei were counterstained with SYTOX Green. Numbers of CD4+ cells were compared between blubber layers using paired t-test. Scale bar is 50 μm.

The two outermost layers of blubber (approx. 2 cm below the epidermis) were defined by high expression of *AGT*, *CD4*, and *COL1A2*, which decreased toward the inner blubber layers (Figure 5). Consistent with this observation, we detected higher numbers of CD4+ cells in outer compared to inner blubber (*t* = 4.35, *p* = 0.012; Figure 6). Positioning within the blubber depth explained 80% of the variance (marginal *R* ^*2*^) in *CD4* gene expression, 67% of the variance in *COL1A2* expression, and 42% of the variance in *AGT* expression. *AGT* expression was highest in layers 1 and 2 and lowest in layers 4 and 5 (*F*_4,16_ = 23.02, *p* < 0.0001; post-hoc tests: *p* < 0.05; Figure 5); it did not vary significantly between layers 1 and 2 or between layers 4 and 5 (*p* > 0.05). *COL1A2* expression was higher in layers 1 and 2 than layers 3–5 (*F*_4,16_ = 18.00, *p* < 0.0001; post-hoc tests: *p* < 0.05; Figure 5); its expression did not vary between layers 3–5 (*p* > 0.05). *CD4* expression was highest in layer 1, decreased significantly between layers 1 and 2, and was lowest in layers 4 and 5 (*F*_4,16_ = 33.92, *p* < 0.0001; post-hoc tests: *p* < 0.05; Figure 5).

The innermost two blubber layers (approx. 2 cm above the muscle) were defined by high expression of *ADIPOQ*, *ANGPTL4*, *CES1*, *GABRG2*, *HCAR2*, *HMGCS2*, *LEP*, and *THBS1*, which decreased toward the outer layers (Figure 5). Positioning within the blubber depth explained 92% of the variance in *GABRG2* and *HMGCS2* expression, 77% of the variance in *HCAR2* expression, 69% of the variance in *ADIPOQ* and *LEP* expression, and 55% of the variance in *ANGPTL4* expression. However, blubber depth explained only 23% of the variance in *THBS1* expression and 8.8% of the variance in *CES1* expression. *GABRG2* and *HMGCS2* expression decreased significantly between each blubber layer from 5 to 1 (*GABRG2*: *F*_4,16_ = 114.67, *p* < 0.0001, post-hoc tests *p* < 0.05; *HMGCS2*: *F*_4,16_ = 136.66, *p* < 0.0001, post-hoc tests *p* < 0.01; Figure 5). *HCAR2* expression was highest in layer 5, decreased from layer 4 to 2, and was lowest in layer 1 (*F*_4,16_ = 31.88, *p* < 0.0001, post-hoc tests: *p* < 0.01). *ANGPTL4* expression was highest in layers 4 and 5, decreased significantly between layers 4 and 2, and was lowest in layers 1 and 2 (*F*_4,16_ = 15.10, *p* < 0.0001, post-hoc tests *p* < 0.05). *THBS1* expression was highest in layers 4 and 5 and lowest in layer 1 (*F*_4,16_ = 6.69, *p* = 0.0023, post-hoc tests: *p* < 0.05), but did not vary significantly among the middle layers (post-hoc tests: *p* > 0.05). *ADIPOQ* and *LEP* expression was highest in layers 3–5, intermediate in layer 2, and lowest in layer 1 (ADIPOQ: *F*_4,16_ = 93.60, *p* < 0.0001, post-hoc tests: *p* < 0.01; LEP: *F*_4,16_ = 48.74, *p* < 0.0001, post-hoc tests: *p* < 0.05; Figure 5); expression of neither gene varied between layers 3–5 (*p* > 0.05). *CES1* expression was significantly higher in layers 2–5 than layer 1 (*F*_4,16_ = 10.85, *p* < 0.001, post-hoc tests: *p* < 0.05), but did not vary between the deeper four layers (*p* > 0.05).

## Discussion

**FIGURE 7:**
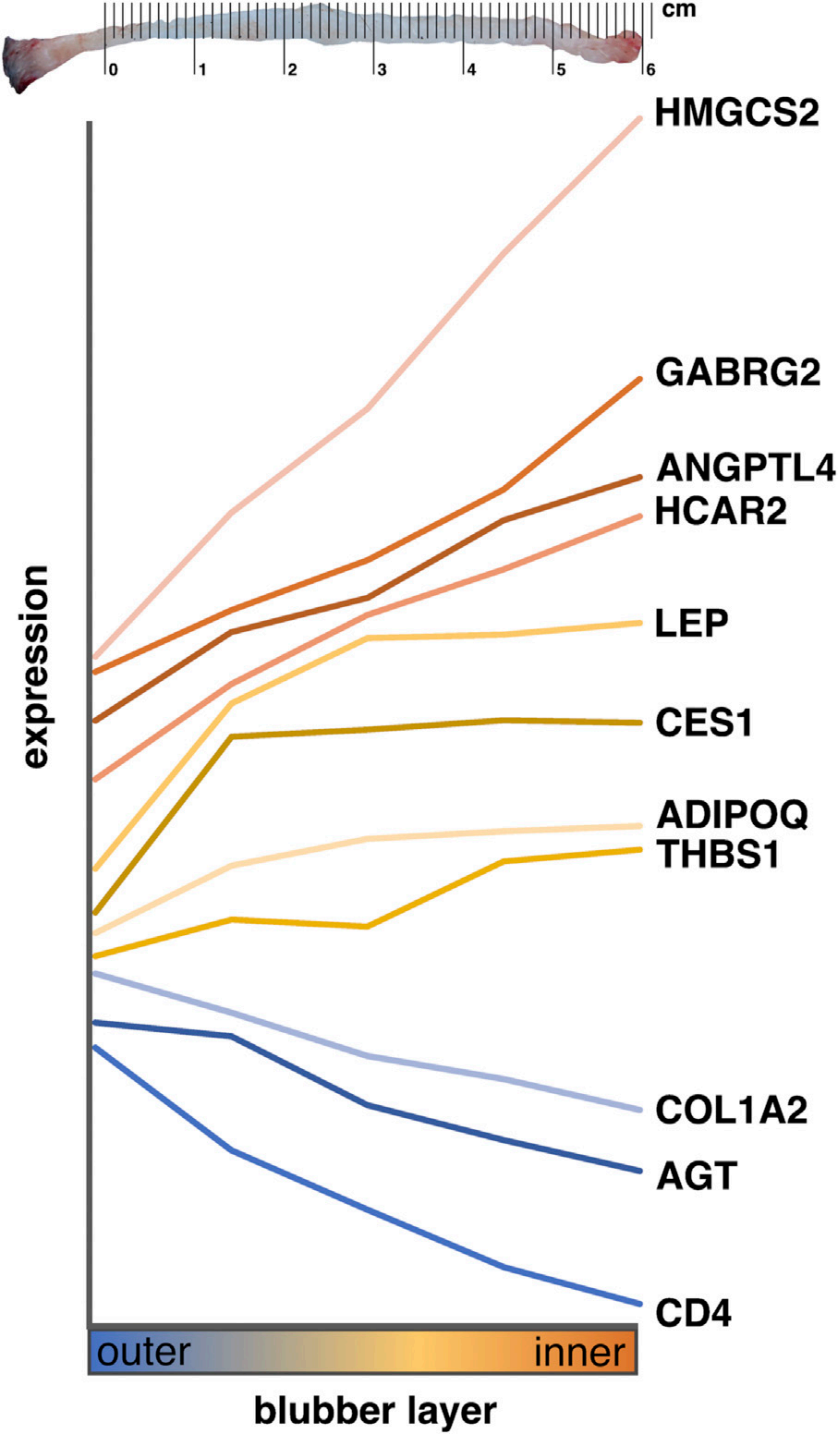
Summary of gene expression across blubber depth in elephant seals. Lines show mean normalized expression levels (delta C_T_) of genes shown in Figure 5 across five blubber layers. Genes are ordered by expression pattern across blubber depth, not absolute expression level. Genes shown in orange were most highly expressed in innermost blubber, those shown in yellow had highest expression in middle and inner blubber layers, and those shown in blue were expressed most highly in outermost blubber. Metric ruler was created using Vector Ruler Generator (MIT License).

This study examined morphological, cellular, and molecular stratification of blubber tissue in the northern elephant seal, a deep-diving phocid that is adapted to prolonged fasting of up to 4 months on land. We collected blubber from juvenile elephant seals (weaned pups and 1–2 year-olds) during the early stage of their fasting periods. We found subtle but significant morphological differences between blubber layers: inner blubber contained adipocytes that were larger and more heterogeneous in size, higher abundance of CD144+ endothelial cells, and lower abundance of CD4+ immune cells than outer blubber. We also identified 61 annotated genes that were differentially expressed between inner and outer blubber layers. Based on the known functions of these genes in other mammals, we suggest that inner blubber has potentially higher 1) adipogenic capacity, 2) cellular diversity, and 3) metabolic and neuroendocrine signaling activity than outer blubber. On the other hand, outer blubber may have higher 1) ECM synthesis activity and 2) responsiveness to pathogens and cell stressors than inner blubber. We further characterized expression of nine genes of interest identified by transcriptomics and two genes encoding adipokines with higher precision using targeted assays of elephant seal blubber tissue divided into five layers across its depth. We found that the inner 1–2 cm of blubber has the highest expression of adipokines and metabolic enzymes, and propose that these genes may be used to molecularly delineate blubber layers (Figure 7). Additionally, their known functions in other species may be used to generate hypotheses about the physiology of this unique tissue in marine mammals.

### Blubber morphology

We found that adipocytes in the inner layer of blubber collected from juvenile elephant seals were larger and more heterogeneous in size than those in outer blubber. Similarly, a previous study of weaned elephant seal pups showed that outer blubber contained a larger proportion of small adipocytes (area <2,800 μm^2^) than inner blubber (Louis et al., 2014). This suggests that adipocytes in inner blubber may have a higher capacity for lipid storage and higher rates of lipid and cell turnover than those in outer blubber. However, this pattern varied between individuals, likely due to the influence of nutritional state and body condition at the time of sampling on adipocyte size and blubber layer thickness. While the juvenile animals used in our study were sampled early in their fasting period and had apparent high adiposity, their date of arrival at the rookery, and therefore the number of days spent fasting prior to sampling, was unknown. In weaned pups, the proportion of large (area 5,600–8,400 μm^2^) and mid-size (area 2,800–5,600 μm^2^) adipocytes decreased, while the proportion of small adipocytes increased over 7 weeks of fasting in inner and outer blubber layers (Louis et al., 2014). Further studies will be necessary to determine whether the change in adipocyte size over fasting differs between blubber layers across life history. While we expected the size of adipocytes in the middle blubber region to be intermediate between that of inner and outer blubber, it was quite variable: in two animals, adipocytes in the middle layer were similar in size to those in outer blubber, whereas in the other two animals, they were larger and more similar in size to those in inner blubber. These findings, together with our gene expression data (discussed below), suggest that blubber composition and function may shift across a gradient, rather than display a clear transition point between layers.

Our adipocyte measurement data were also consistent with those from a recent comparative study of marine mammal blubber that found larger adipocytes in deep compared to superficial blubber in harbor seal, minke whale, Risso’s dolphin, and Blainsville’s, Sowerby’s, and Cuvier’s beaked whales (Gabler-Smith et al., 2022). In contrast, adipocytes were larger in superficial compared to deep blubber in California sea lion, Atlantic spotted dolphin, and Gervais’s beaked whale. While Gabler-Smith et al. hypothesized that dive duration influenced adipocyte size and blubber vascularization, the former is also likely to be affected by life history strategy (i.e., income vs. capital breeding; Champagne et al., 2012). However, the life history stages at which the animals in Gabler-Smith’s study were sampled were unknown, and further studies will be necessary to confirm these findings. The data from our and Gabler-Smith’s study highlight the influence of both phylogeny (e.g., phocid vs*.* otariid) and life history strategy (e.g., adaptation to fasting) on blubber morphology.

Our immunolabeling data using the endothelial marker CD144 confirmed our hypothesis that inner blubber of northern elephant seals is more highly vascularized than outer blubber. This finding was consistent with studies showing significantly or marginally higher microvascular density in inner relative to outer blubber of bottlenose dolphins, harbor seals, and California sea lions (McClelland et al., 2012; Gabler-Smith et al., 2022), although neither study used cell-specific markers. The higher vascular density of inner blubber suggests that this layer may be more responsive to endocrine signals, such as those that promote adipogenesis and stimulate lipolysis, than outer blubber—a hypothesis that was supported by our transcriptome data.

### Genes associated with adipogenesis

Multiple genes upregulated in inner relative to outer blubber were associated with adipogenesis, the process by which mesenchymal stem cells (MSC) become committed to the adipocyte lineage (i.e., become pre-adipocytes) and then terminally differentiate into mature adipocytes. This process establishes adipose tissue during early development, supports adipocyte turnover throughout adulthood, and enables animals to respond to rapid increases in nutrient intake and other stimuli (Rigamonti et al., 2011). Adipogenesis is regulated by a number of factors, including the Wnt signaling pathway, glucocorticoids, and insulin (Ghaben and Scherer, 2019). Our previous work in elephant seals showed that several pro-adipogenic genes (*DKK1*, *KLF15*, *CEBPD*, and *PPARG*) are upregulated in inner blubber in response to elevated glucocorticoids, but we did not assess their expression in outer blubber (Khudyakov et al., 2017; Deyarmin et al., 2019).

In this study, we found that four members of the Wnt signaling pathway—*WNT6*, *SFRP1*, *FRZB*, and *DKK2*—had higher expression in inner blubber compared to outer blubber. Wnt ligands, including Wnt 6, are known inhibitors of adipogenesis (Ghaben and Scherer, 2019), and may function to maintain a pool of multipotent MSCs, in part by inhibiting apoptosis (Longo et al., 2002). Levels of the anti-apoptotic Bcl-2 protein have been shown to increase during adipogenesis in mice (Sorisky et al., 2000); accordingly, the most highly upregulated gene in inner relative to outer blubber in this study was *BCL2A1*, another member of the BCL2 family. *SFRP1*, *FRZB* (also known as *SFRP3*), and *DKK2* are secreted inhibitors of Wnt (Guan et al., 2021). While SFRP1 promotes adipogenesis, DKK2 was recently shown to inhibit adipocyte differentiation (Yang and Shi, 2021). Co-expression of both inducers and inhibitors of adipogenesis in inner blubber suggests that this tissue contains a developmentally active, heterogenous population of cells in various stages of commitment to the adipocyte lineage, including pools of proliferating MSCs and pre-adipocytes as well as terminally differentiated adipocytes. In contrast, outer blubber may contain a larger proportion of quiescent, mature adipocytes that store fat for insulation. Our morphological data support these ideas, as adipocytes in outer blubber were more homogeneous in size than those in inner blubber. Further studies will be necessary to determine whether the proportion of mitotic cells varies between blubber layers.

### Genes associated with metabolism and neuroendocrine signaling

Based on the prevalence of dietary fatty acids and higher degree of vascularization in inner blubber, it has long been hypothesized that this layer is more metabolically active and more responsive to neuroendocrine signals than outer blubber (Derous et al., 2020). A recent *in vitro* study showed that inner blubber of elephant seals produces more leptin than outer blubber and that the dynamics of glycerol release in response to lipolytic stimuli vary with fasting duration in inner, but not outer blubber (Debier et al., 2020). Our gene expression data support these hypotheses: expression of genes associated with lipolysis, lipid uptake into adipocytes, ketogenesis, and lipogenesis (*CES1*, *ANGPTL4*, *CCK1*, *HMGCS2*, *HCAR2*, and *ADRB1*) was higher in inner relative to outer blubber. Two of these genes encode hormones (*ANGPTL4*, *CCK*), while another encodes a receptor for hormones and neurotransmitters (*ADRB1*). Functional studies will be necessary to definitively confirm that neuroendocrine signaling activity is higher in inner relative to outer blubber.

We did not detect differences in expression of genes encoding the intracellular lipases ATGL and HSL between blubber layers, as previously reported in elephant seal pups (Louis et al., 2015). However, expression of carboxylic ester hydrolase *CES1*, a lipase that hydrolyzes triglycerides and cholesteryl esters, was significantly higher in inner compared to outer blubber. *CES1* is associated with lipid droplets and is involved in basal lipolysis in mice (Grabner et al., 2021). In humans, *CES1* is thought to be a marker of adipocyte lipid content as it is more highly expressed in large compared to small adipocytes (Jernås et al., 2009). Our findings were consistent with this study as adipocytes in inner blubber, which expresses high levels of *CES1*, were larger than those in outer blubber in three of four individual animals. *ANGPTL4* encodes angiopoietin-like 4, an adipokine that inhibits lipoprotein lipase (LPL), thus reducing triglyceride uptake and storage by adipocytes. Its abundance increases during the fasted state in humans and rodents and it is considered a critical regulator of the switch between fed and fasted states (Kersten, 2021)—a key feature of metabolism of many marine mammal species. High expression of *ANGPTL4* in inner blubber may contribute to the low LPL activity previously reported in adult female elephant seals (McDonald and Crocker, 2006). *ANGPTL4* expression is regulated by glucocorticoids, which increase over fasting in elephant seals, and may contribute to insulin resistance, which is also displayed by fasting elephant seals (Crocker et al., 2014; Chen et al., 2017). Therefore, ANGPTL4 may serve as a critical regulator of fasting metabolism in fasting-adapted marine mammals.

*CCK* encodes cholecystokinin, a hormone primarily secreted by endocrine cells within the small intestine in response to peptides and lipids leaving the stomach. Recent studies have shown that *CCK* is also expressed by adipocytes in rats and humans (Plaza et al., 2018b), where it promotes fatty acid uptake and triglyceride storage by inhibiting ANGPTL4 (thereby activating LPL) and stimulating the release of adiponectin (Plaza et al., 2018a). Co-expression of both activators and inhibitors of fat uptake and storage in inner blubber suggests that this layer retains a fine-tuned sensitivity to metabolic stimuli in elephant seals.

*HMGCS2* encodes 3-hydroxy-3-methylglutaryl-CoA synthase 2, the first rate-limiting enzyme in the ketogenesis pathway that also regulates fatty acid beta-oxidation in the liver (Vila-Brau et al., 2011). Our previous work identified *HMGCS2* as one of the principal stress-regulated genes in elephant seal blubber (Khudyakov et al., 2017; Deyarmin et al., 2019; Pujade Busqueta et al., 2020), but its expression and function in adipose tissue of other mammals has not been studied until very recently. It has now been shown that *HMGCS2* is expressed by mature white adipocytes in mouse, which secrete the ketone body 3-hydroxybutyrate (BHB), that, in turn, stimulates expression of genes encoding antioxidant and lipogenic enzymes and adipokines (Nishitani et al., 2022). *HMGCS2* was also shown to be one of the most highly upregulated genes in human BAT compared to WAT, where it stimulates thermogenesis *via* production of mevalonate (Balaz et al., 2019). Interestingly, another gene upregulated in inner blubber, *HCAR2*, encodes hydroxycarboxylic acid receptor 2, a nutrient-sensing receptor that binds BHB and inhibits lipolysis, as well as stimulating adiponectin release during fasting (Rojas-Morales et al., 2016). This presents the intriguing hypothesis that inner blubber may be a local source of ketones in marine mammals, which may act as paracrine regulators of lipid metabolism in blubber (and potentially underlying skeletal muscle), and contribute to the high antioxidant capacity of animals adapted to prolonged breath-holds (Allen and Vázquez-Medina, 2019).

In contrast to inner blubber, outer blubber had higher expression of only three notable genes associated with fat metabolism (*AGT*, *CYP1B1*, and *F12A1*). *AGT* encodes angiotensinogen, a component of the renin-angiotensin-aldosterone system (RAAS) that is primarily produced by the liver. Recent studies in both humans and mice found that all components of the RAAS are expressed and produced locally by white adipose tissue, and that *AGT* overexpression in mouse adipocytes increased lipid content and fatty acid synthase expression (Frantz et al., 2018). *CYP1B1* is involved in metabolism of steroids and fatty acids; its knockdown in mice reduces development of obesity in response to a high-fat diet (Liu et al., 2015). *F13A1* encodes transglutaminase FXIII-A, which is associated with adipose tissue hypertrophy and expression of components of cell stress and tissue remodeling pathways in humans (Kaartinen et al., 2021). *F13A1* expression is negatively associated with adiponectin in humans, consistent with our findings that *F13A1* was more highly expressed in outer blubber, while *ADIPOQ* was more highly expressed in inner blubber of seals. Higher expression of *AGT*, *CYP1B1*, and *F13A1* in outer blubber suggests that this layer may function primarily to store fat for insulation, rather than mobilizing it in response to endocrine signals.

### Genes associated with cellular diversity

White adipose tissue of terrestrial mammals has been shown to contain a diverse array of cell types besides white adipocytes, including MSCs, beige/brite adipocytes, immune cells (e.g., macrophages, T cells), vascular cells (endothelial and smooth muscle cells), and fibroblasts (Lenz et al., 2020). Several genes with higher expression in inner relative to outer blubber (*THBS1*, *FMOD*, *DKK2*, *GABRG2*, and *ADRB1*) were associated with these other cell types and may thus reflect differences in cellular heterogeneity across blubber depth. *THBS1* is an adipokine produced by adipocytes, endothelial cells, smooth muscle cells, and fibroblasts within adipose tissue, and has been shown to inhibit angiogenesis and adipocyte browning in humans (Gutierrez and Gutierrez, 2021). *FMOD* is expressed by MSCs, fibroblasts, and myocytes and has been shown to stimulate angiogenesis and myogenesis (Lee et al., 2018). The co-expression of both activators and inhibitors of angiogenesis suggests that this process is intricately regulated within inner blubber tissue.

*DKK2*, *GABRG2*, and *ADRB1* have been associated with browning or “beiging” of white adipose tissue in other mammals, a process by which adipocytes residing within white adipose tissue depots acquire thermogenic capacity, a hallmark of brown adipocytes (Cohen and Kajimura, 2021). *DKK2* is highly expressed in beige adipocytes in mice and was recently proposed as a novel beige fat adipokine (Yang and Shi, 2021). *GABRG2* encodes a receptor for the neurotransmitter GABA, which has been detected, along with several other GABA receptors, in mouse brown adipose tissue (Ikegami et al., 2018). *GABRG2* is also highly expressed in small proliferating adipocytes of mice, which were suggested to be potential beige adipocyte progenitors (Taguchi et al., 2020). *ADRB1* encodes the beta-adrenergic receptor for catecholamines, which stimulate lipolysis and induce adipocyte browning and thermogenesis (Collins, 2022). These findings suggest the possibility that some adipocytes within inner blubber tissue may have thermogenic capacity. However, while UCP1, the canonical marker of brown adipocytes, has been detected in cetacean blubber (Hashimoto et al., 2015), we have not been able to detect its expression in this or our previous transcriptomes of elephant seal blubber. To date, the question of whether brown adipocytes are present in pinniped blubber remains unresolved.

Several genes upregulated in outer relative to inner blubber (*IL1RL1*, *CD4*, *PBK*) and immunofluorescence staining suggest that the outer blubber layers may have a higher proportion of T cells and possibly other immune cells than inner layers. The most highly upregulated gene in outer blubber was *IL1RL1*, which is expressed primarily by regulatory T cells, helper T cells, and eosinophils in human and mouse adipose tissue. IL1RL1 is the receptor for IL-33, a ligand produced by fibroblasts, endothelial cells, and macrophages in adipose tissue in response to infection or trauma (Mahlakõiv et al., 2019). CD4 is the ligand for the T cell receptor expressed by helper T cells, regulatory T cells, macrophages, and monocytes within adipose tissue of mice and humans (Wang and Wu, 2018). *PBK* encodes lymphokine-activated killer T-cell-originated protein kinase, which is expressed by activated cytotoxic T cells, other lymphoid cells, and other rapidly proliferating cell types (Abe et al., 2000). Immune cells in white adipose tissue have been studied primarily in the context of their role in adipose tissue inflammation during obesity in humans. Expression of markers of regulatory T cells (*IL1RL1*, *CD4*), which restrain inflammation, in blubber of seals suggests a potential mechanism for prevention of adipose tissue inflammation in naturally “obese” mammals. Adipose-associated immune cells likely contribute to tissue homeostasis in other ways. For example, they may provide protection against pathogens that infiltrate adipose tissue, regulate adipocyte responses to nutrients, and participate in remodeling of the ECM surrounding adipocytes (Man et al., 2017). Accordingly, adiposity is the primary driver of circulating inflammatory cytokine concentrations in female elephant seals (Peck et al., 2016). Together, these findings are the first to report the presence of transcriptionally active immune cells in marine mammal blubber.

### Genes associated with extracellular matrix and responses to pathogens and cellular stress

Outer blubber had significantly higher expression of genes associated with production of ECM proteins (*CREB3L1*, *COL1A1*, *COL1A2*, and *GPC1*) than inner blubber. CREB3L1 is involved in cellular secretion of collagen, *GPC1* encodes glypican-1, and *COL1A1* and *COL1A2* encode components of type I collagen, the most abundant component of dermal ECM. These expression data are consistent with the hypothesis that outer blubber has a structural function and histological studies in cetaceans showing higher densities of ECM fibers in outer relative to inner blubber (Struntz et al., 2004; Montie et al., 2008). Lastly, in accordance with its location closer to the surface of the animal, which is exposed to pathogens and environmental stressors, outer blubber also had higher expression of genes associated with innate (*CFB*) and adaptive immunity (*CD4*, *IL1RL1*, *PBK*) and response to cold (*STMN1*) and cellular stress (*HSPE1*, *PBK*, and *PARP4*) compared to inner blubber.

### Gene expression signature for delineating blubber layers

Our targeted assays showed that expression of nine of the eleven genes we investigated—*ADIPOQ*, *AGT*, *ANGPTL4*, *CD4*, *COL1A2*, *GABRG2*, *HCAR2*, *HMGCS2*, and *LEP*—may be used to molecularly distinguish inner from outer blubber (Figure 7). The remaining two genes—*CES1* and *THBS1*—did not delineate inner from outer blubber at high resolution: *THBS1* expression was highly variable between individuals, while *CES1* expression only differed between the outermost and deeper four layers. The innermost ∼2 cm of blubber (layers 4 and 5) were defined by high expression of the metabolic enzyme *HMGCS2*, nutrient sensor *HCAR2*, GABA receptor *GABRG2*, and adipokines *ANGPTL*, *ADIPOQ*, and *LEP*, and low expression of the RAAS component *AGT*, T cell marker *CD4*, and ECM component *COL1A2*. In contrast, the outer ∼2-cm of blubber were defined by high expression of *AGT*, *CD4*, and *COL1A2* and low expression of the other markers. We also confirmed that expression of two well-studied adipokine genes, *LEP* and *ADIPOQ*, was higher in inner compared to outer blubber. This was consistent with a study of *LEP* expression in cetacean blubber and higher leptin protein production observed in cultured slices of elephant seal blubber (Ball et al., 2017; Debier et al., 2020).

## Conclusion and caveats

This study was the first to examine blubber stratification at transcriptome-level resolution in any marine mammal and to assess differences in morphology between blubber layers in the northern elephant seal. We showed that immunohistochemistry using cell specific markers (CD144, CD4) may be used to detect endothelial and resident immune cells in marine mammal blubber, and that RT-qPCR targeting a handful of genes may be used to delineate inner from outer blubber. Our data suggest that inner blubber of elephant seals is more highly vascularized, contains a more variable adipocyte population, and has higher expression of genes associated with lipid storage and mobilization, adipocyte differentiation, and neuroendocrine signaling. Based on our data, we suggest that researchers interested in metabolic physiology of pinnipeds should target the innermost 1–2 cm of blubber closest to skeletal muscle in their studies. Outer blubber, however, was not transcriptionally inactive, displaying higher expression of genes associated with ECM remodeling, immune signaling, and responses to cell stressors than inner blubber. While we used animals of different age classes for transcriptome and RT-qPCR analyses (juveniles and weaned pups, respectively), the gene expression patterns identified by both approaches were remarkably similar, suggesting that molecular stratification of blubber may be conserved across life history in elephant seals. However, the morphological profiles and gene expression patterns described here were obtained from young animals sampled at the beginning of their fasting periods, and further work will be necessary to determine whether they vary with age, sex, and prolonged fasting.

## Data Availability

Raw RNA sequencing data are available at the NCBI Sequence Read Archive (BioProject ID: PRJNA874098). Blubber transcriptome assembly and annotation are available at Figshare (https://doi.org/10.6084/m9.figshare.21097804.v1).
